# The Association of the Metabolic Score for Insulin Resistance (METS-IR) with Prehypertension in Normoglycemic Individuals

**DOI:** 10.5812/ijem-145894

**Published:** 2024-08-03

**Authors:** Methaq H Alogaili, Afnan A Alsallami

**Affiliations:** 1 Deptartment of Family and Community Medicine, College of Medicine, Al-Nahrain University, Baghdad, Iraq; 2 College of Medicine, Al-Nahrain University, Baghdad, Iraq

**Keywords:** Cross-Sectional Study, Insulin Resistance, METS-IR, Prehypertension

## Abstract

**Background:**

Insulin resistance is a critical first step in the development of many chronic diseases such as diabetes, hypertension, and coronary artery disease (CAD). Early recognition of changes in insulin sensitivity and subsequent glycolipid dysregulation is paramount in reversing the progression of these diseases. Since the prevalence of insulin resistance is relatively high, there is a demand for a simple, easy, and inexpensive method for its detection.

**Objectives:**

To assess the association of metabolic score for insulin resistance (METS-IR) with prehypertension (preHTN) in normoglycemic individuals.

**Methods:**

A cross-sectional study included two groups with normal blood glucose levels (age and sex matched, 179 adults each) with and without preHTN. Those with a history of hypertension, diabetes, CAD, or on chronic drug treatment were excluded from the study. Metabolic score for insulin resistance was calculated as Ln [(2 × FPG (mg/dL) + fasting TG (mg/dL)] × BMI (kg/m²)/Ln [HDL-c (mg/dL)].

**Results:**

The METS-IR score was significantly higher in those with preHTN (P < 0.001). One-way ANOVA showed significant differences in BMI, TG, and HDL-c according to METS-IR quartiles. Cox regression analysis showed that METS-IR is a single independent predictor of preHTN (OR = 1.3, 95% CI 1.20, 1.34).

**Conclusions:**

Metabolic score for insulin resistance is significantly associated with and an independent predictor of prehypertension in euglycemic people.

## 1. Background

Insulin resistance (IR), or decreased insulin sensitivity, is defined as ineffective insulin activity in peripheral tissues, leading to a subsequent state of hyperinsulinemia with impaired lipid and glucose regulation (1). Insulin resistance is a risk factor for the development of type 2 diabetes mellitus (T2DM). Along with central obesity, systemic hypertension (HTN), and dyslipidemia—characterized by high triglycerides (TG) and low levels of high-density lipoprotein cholesterol (HDL-c)—IR forms the basis for metabolic syndrome, or syndrome X (2). Recent studies have documented that IR contributes to the development of progressive vascular stiffness and higher blood pressure values, independent of hyperlipidemia or hyperglycemia (3). Furthermore, IR is associated with accelerated atherosclerosis due to chronic inflammation and vascular endothelial dysfunction, significantly increasing cardiovascular (CV) risk (4).

Among adults, the global prevalence of IR ranges from 17 to 51%, with higher rates in low and middle-income countries (5). The relationship between elevated blood pressure and these complications is continuous and begins as early as an office SBP >115 mmHg and a DBP > 75 mmHg. Thus, the cut-off values that define "normal" blood pressure are somewhat arbitrary (6, 7). The clinical importance of HTN lies in two vital areas: First, CV diseases are the most common non-communicable diseases (NCD) worldwide; second, CV events related to HTN and its metabolic consequences are the leading causes of death from NCDs globally (8). Insulin resistance has a tremendous impact on the pathophysiology of HTN and the subsequent development of CV diseases and complications, which are further exacerbated by the presence of lipid and glucose abnormalities (1).

Prehypertension (preHTN), defined as SBP 120 to 139 mmHg and DBP 80 to 89 mmHg, is the intermediate stage between HTN and normal blood pressure that may signal the development of HTN (8). Prehypertension affects about 25 - 50% of adults worldwide. However, due to the silent nature of preHTN, its prevalence is underreported, especially in low and middle-income countries (9). Studies have shown that preHTN carries health risks nearly comparable to those with established HTN (10-12). These risks, although not always clinically evident, are related to a collection of metabolic disturbances that form a continuum of metabolic syndrome (2). At the root of this complex metabolic network is IR. The hyperinsulinemic euglycemic clamp (HEC), traditionally considered the gold-standard method to assess insulin sensitivity in vivo, is invasive, time-consuming, costly, and somewhat difficult to perform, especially in primary health care (PHC) settings (13).

The new noninvasive metabolic score for insulin resistance (METS-IR) provides an easy, less expensive, and suitable method for routine clinical use compared to the standard HEC. This method is especially advantageous in low and middle-income countries for revealing IR and is more correlated with HEC than other non-insulin-based methods (14). Early detection of IR is crucial to halting disease progression, enabling abnormality reversion, and reducing morbidity, mortality, and the public health burden. Recent studies have demonstrated an association between METS-IR and various chronic diseases, predicting the risk of complications and mortality (14, 15). However, no study has explored the relationship between METS-IR and preHTN in Iraq. This study is among the very few to investigate the association of METS-IR among euglycemic prehypertensive individuals, making a comparison with those with normal blood pressure.

## 2. Objectives

To highlight the association of METS-IR in people with preHTN who have normal glucose levels and to explore some associated demographic characteristics.

## 3. Methods

This cross-sectional study included 179 adults with preHTN and normal glucose levels who were individually matched (age and sex) on a 1:1 basis to a comparison group with normal blood pressure and normal glucose levels. Participants were recruited from twelve PHC centers distributed equally throughout the capital of Iraq, Baghdad. A multistage random sampling technique was used to select PHC centers from all health sectors of both the Kerkh and Rusafa directorates. Study participants were all adults aged 21 - 79 years. A direct interview was conducted with each participant to gather the required information, including a complete medical and family history and medication use. Those with a history of HTN, T2DM, CV disease, or chronic drug treatment were excluded from the study. Weight was measured to the nearest 0.5 kg using an electronic scale, with participants in an erect position without shoes and wearing light clothing. Height was measured using a height tape with an approximation of ± 0.1 cm, and Body Mass Index (BMI) was calculated as body weight/height² (kg/m²). Blood pressure was measured using a mercury sphygmomanometer on the participant’s arm in a sitting position. Two blood pressure readings were taken at 10-minute intervals, and the mean value for both systolic and diastolic pressure was calculated in millimeters of mercury (mmHg). Blood was drawn from each participant after confirmation of 8 - 12 hours of overnight fasting. Two milliliters were collected in a vacuum collection K3 EDTA tube, mixed thoroughly, and analyzed by a laboratory technician on the same day. A Siemens Dimension EXL 200 was used to measure serum fasting blood glucose (FPG) concentrations, TG, and HDL-c. Prehypertension was defined as systolic pressure from 120 – 139 mmHg or diastolic pressure from 80 – 89 mmHg (6). Diabetes mellitus (DM) was defined as a fasting plasma glucose (FPG) level ≥ 126 mg/dL, HbA1c > 6.4%, or the use of oral hypoglycemic agents or insulin (16). Metabolic score for insulin resistance was calculated as {Ln [(2 × FPG (mg/dL) + fasting TG (mg/dL)] × BMI (kg/m²)/Ln [HDL-c (mg/dL)]} (14).

### 3.1. Data Analysis

Continuous variables were presented as mean ± standard deviation (SD). A paired *t*-test was used to compare the mean differences between the two groups, and one-way ANOVA was used to compare the mean differences between the METS-IR quartiles of the preHTN group. Categorical variables were expressed as counts and frequencies, and the chi-square test was used to analyze the association between preHTN and the comparison group data. The Pearson correlation coefficient was used to evaluate the association between METS-IR and risk factors for the entire study population. Cox regression analysis was used to predict METS-IR in the preHTN and comparison groups, with adjustment for other covariates. The time variable was set to 1 for "case" and 2 for "control." Since the traditional logistic model does not explicitly account for changes in covariate values over time (and many exposures are subject to variation over time), the Cox regression model was utilized instead (17, 18).

Data were entered and analyzed using SPSS (statistical package for social sciences), version 25, and a P-value of less than 0.05 was considered significant. The study was approved by the scientific and research committee of the Family and Community Department at the College of Medicine, Al-Nahrain University, and a request to facilitate the task of the researcher was granted. Participants were directly interviewed and informed about the research purpose and procedure. Their acceptance to participate was obtained, and confidentiality of their information was ensured.

## 4. Results

The mean age of the participants was 44.9 ± 12.0 years (range 21 - 65 years), with a mean BMI of 29.6 ± 3.8. Males comprised 61.5% of the participants. There was a significant difference in the mean values of BMI, SBP, DBP, FBG, TG, and HDL-c between the two groups (Table 1). 

The mean METS-IR score for the preHTN group (46.6 ± 6.4; range 34.1 - 61.2) was significantly higher than that of the comparison group (38.4 ± 4.4; range 29.4 - 51.0) (P < 0.001). When preHTN participants were divided into four groups based on the quartiles of METS-IR (quartile 1 < 37.3, N = 9; 37.3 ≤ quartile 2 < 41.1, N = 37; 41.1 ≤ quartile 3 < 47.0, N = 49; quartile 4 ≥ 47.0, N = 84), one-way ANOVA showed a statistically significant difference in the mean values of BMI, TG, and HDL-c (P-values < 0.001, 0.003, and < 0.001, respectively) (Table 2). In the comparison group, one-way ANOVA showed a statistically significant difference in the mean values of BMI, FPG, TG, and HDL-c (P-values < 0.001, 0.001, 0.006, and < 0.001, respectively) (Table 3). 

**Figure 1.**
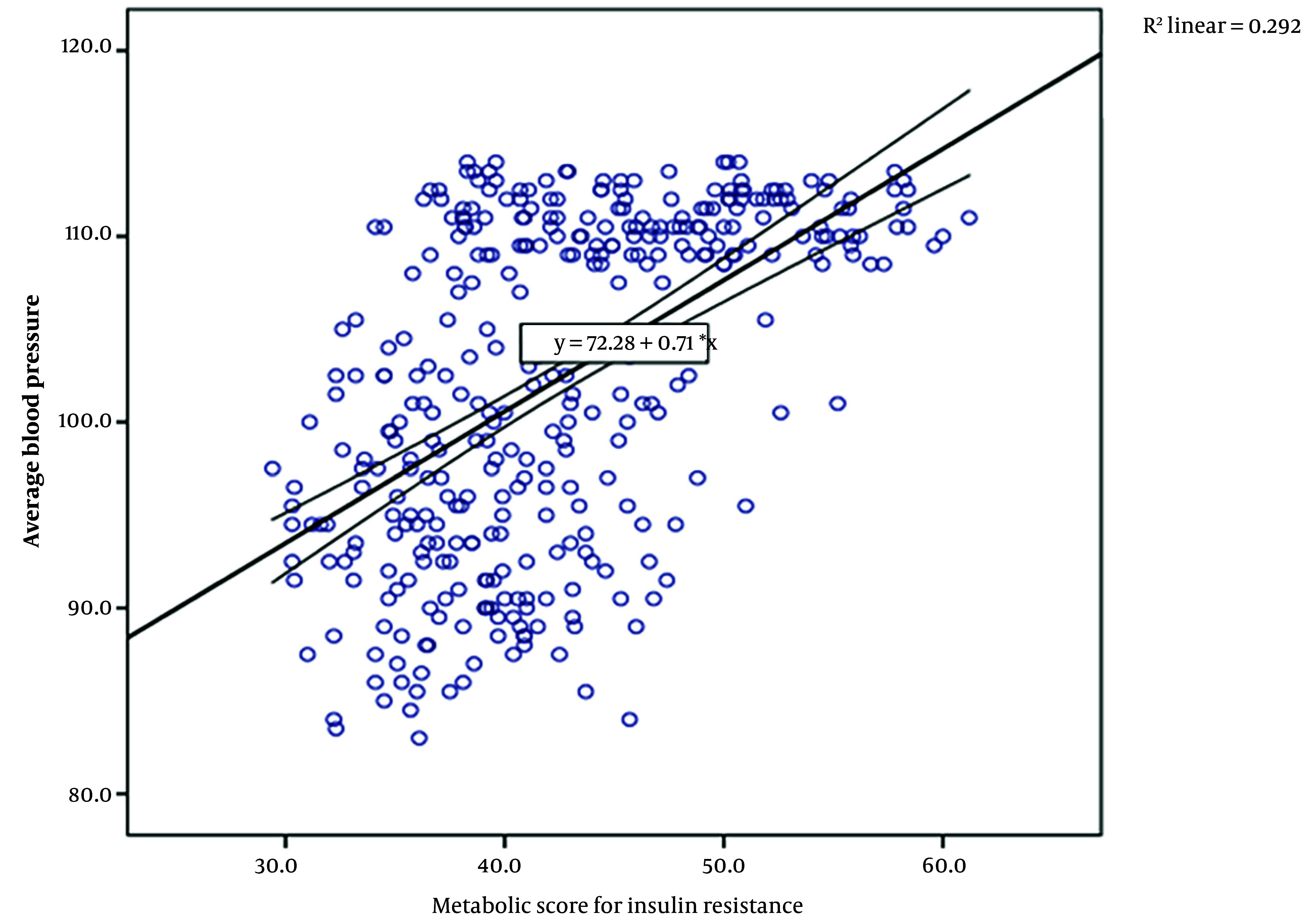
Pearson correlation between metabolic score for insulin resistance (METS-IR) and average blood pressure

The Pearson correlation coefficient showed a significant positive correlation between the METS-IR score and all other risk factors, as well as a significantly positive correlation with the average blood pressure of the two groups (r = 0.54, P < 0.001) (Table 4 and Figure 1). 

Cox regression analysis indicated that METS-IR is an independent predictor of preHTN (OR = 1.3, 95% CI 1.20, 1.39, P < 0.001). Adding other covariates did not improve the overall model (Table 5). 

**Table 1.** Baseline Characteristics of the Study Populations ^a^

Variables	Prehypertension	Normal	P-Value ^b^
**Age (y)**	44.9 ± 12.0	45.0 ± 11.9	0.56
**Male **	110 (61.5)	110 (61.5)	-
**BMI (Kg/m** ^**2**^ **)**	29.2 ± 3.7	26.1 ± 2.4	< 0.001
**SBP (mmHg)**	134.1 ± 4.1	114.2 ± 9.1	< 0.001
**DBP (mmHg)**	86.4 ± 3.6	74.6 ± 5.5	< 0.001
**FBG (mg/dL)**	97.7 ± 5.8	83.4 ± 8.5	< 0.001
**TG (mg/dL)**	158.8 ± 69.0	138.8 ± 47.3	0.03
**HDLc (mg/dL)**	40.3 ± 6.7	50.4 ± 11.3	< 0.001
**METS-IR**	46.6 ± 6.4	38.4 ± 4.4	< 0.001

Abbreviations: METS-IR, metabolic score for insulin resistance; BMI, Body Mass Index; SBP and DBP, systolic and diastolic blood pressure; FPG, fasting plasma glucose; TG, triglyceride; HDL-C, high-density lipoprotein-cholesterol.

^a^ Values are expressed as No (%) or mean ± SD.

^b^ P-value obtained by one way ANOVA test for continuous variables and chi-square test for categorical variables.

**Table 2.** Baseline Characteristics in People with Prehypertension Based on Quartiles of Metabolic Score for Insulin Resistance ^a^

Variables	Quartile 1 (N = 9)	Quartile 2 (N = 37)	Quartile 3 (N = 49)	Quartile 4 (N = 84)	P-Value ^b^
**METS-IR**	35.9 ± 1.1	39.1 ± 1.1	43.8 ± 1.6	52.3 ± 3.6	< 0.001
**Age (y)**	47.4 ± 12.8	44.2 ± 12.5	44.6 ± 11.4	45.9± 12.3	0.91
**Male **	7 (77.8)	25 (67.6)	29 (29.2)	49 (58.3)	0.56
**BMI (kg/m** ^**2**^ **)**	24.3 ± 1.5	24.6 ± 1.1	28.2 ± 2.2	32.4 ± 2.0	< 0.001
**SBP (mmHg)**	134.9 ± 2.1	133.6 ± 4.3	133.8 ± 4.6	134.3 ± 4.0	0.71
**DBP (mmHg)**	85.4 ± 4.2	86.8 ± 3.7	85.8 ± 5.0	86.7 ± 2.3	0.36
**FBG (mg/dL)**	94.4 ± 9.6	97.6 ± 5.6	97.5 ± 4.8	98.5 ± 6.0	0.22
**TG (mg/dL)**	115.7 ± 34.0	131.5 ± 45.2	159.4 ± 76.5	174.4 ± 71.1	0.003
**HDLc (mg/dL)**	49.2 ± 9.9	38.6 ± 5.0	42.2 ± 9.3	39.0 ± 3.6	< 0.001

Abbreviations: METS-IR, metabolic score for insulin resistance; BMI, Body Mass Index; SBP and DBP, systolic and diastolic blood pressure; FPG, fasting plasma glucose; TG, triglyceride; HDL-C, high-density lipoprotein-cholesterol.

^a^ Values are expressed as No (%) or mean ± SD.

^b^ P-value obtained by one way ANOVA test for continuous variables and chi-square test for categorical variables.

**Table 3.** Baseline Characteristics in People with Normal Blood Pressure Based on Quartiles of Metabolic Score for Insulin Resistance ^a^

Variables	Quartile 1 (N = 79)	Quartile 2 (N = 54)	Quartile 3 (N = 40)	Quartile 4 (N = 6)	P-Value ^b^
**METS-IR**	34.5 ± 2.0	39.3 ± 1.1	43.6 ± 0.26	48.3 ± 1.4	< 0.001
**Age (y)**	44.7 ± 12.8	46.4 ± 11.1	45.0 ± 11.0	36.3 ± 10.1	0.27
**Male **	50 (63.3)	29 (53.7)	26 (65.0)	5 (83.3)	0.41
**BMI (kg/m** ^**2**^ **)**	24.2 ± 1.5	27.0 ± 1.7	28.0 ± 1.8	30.4± 1.6	< 0.001
**SBP (mmHg)**	114.6 ± 9.0	112.1 ± 8.5	115.6 ± 9.7	118.8± 7.1	1.42
**DBP (mmHg)**	74.2 ± 5.7	74.9 ± 5.5	75.0 ± 5.3	74.8± 5.0	0.84
**FBG (mg/dL)**	80.7 ± 7.7	85.4.6 ± 8.6	85.1 ± 8.5	88.7 ± 4.2	0.001
**TG (mg/dL)**	129.3 ± 43.1	135.4 ± 49.9	159.8 ± 45.4	156.1 ± 52.4	0.006
**HDLc (mg/dL)**	54.8 ± 9.7	51.3 ± 11.1	42.2 ± 9.4	38.2 ± 3.7	< 0.001

Abbreviations: METS-IR, metabolic score for insulin resistance; BMI, Body Mass Index; SBP and DBP, systolic and diastolic blood pressure; FPG, fasting plasma glucose; TG, triglyceride; HDL-C, high-density lipoprotein-cholesterol.

^a^ Values are expressed as No (%) or mean ± SD.

^b^ P-value obtained by one way ANOVA test for continuous variables and chi-square test for categorical variables.

**Table 4.** Correlations Between the Metabolic Score for Insulin Resistance and Risk Factors for the Entire Study Population

Variables	Correlation Coefficient (r)	P-Value
**BMI (kg/m** ^**2**^ **)**	0.91	< 0.001
**SBP (mmHg)**	0.49	< 0.001
**DBP (mmHg)**	0.49	< 0.001
**FBG (mg/dL)**	0.52	< 0.001
**TG (mg/dL)**	0.36	< 0.001
**HDLc (mg/dL)**	-0.51	0.03

Abbreviations: METS-IR, metabolic score for insulin resistance; BMI, Body Mass Index; SBP and DBP, systolic and diastolic blood pressure; FPG, fasting plasma glucose; TG, triglyceride; HDL-C, high-density lipoprotein-cholesterol.

**Table 5.** Cox Regression Analysis for Metabolic Score for Insulin Resistance in Prehypertension and Comparison Group

Variables	df	P-Value	OR	95.0% CI for OR	
**METS**	1	0.000	1.30	1.20	1.39
**BMI (Kg/m** ^**2**^ **)**	1	0.764	1.35	0.19	9.51
**SBP (mmHg)**	1	0.009	1.09	1.02	1.17
**DBP (mmHg)**	1	0.027	1.12	1.01	1.25
**FBG (mg/dL)**	1	0.235	1.06	0.96	1.16
**TG (mg/dL)**	1	0.784	1.00	0.98	1.03
**HDLc (mg/dL)**	1	0.703	0.95	0.70	1.27

Abbreviations: METS-IR, metabolic score for insulin resistance; BMI, Body Mass Index; SBP and DBP, systolic and diastolic blood pressure; FPG, fasting plasma glucose; TG, triglyceride; HDL-C, high-density lipoprotein-cholesterol.

## 5. Discussion

The essence of this study was to evaluate the level of IR in people with normal glucose homeostasis who are at an intermediate stage between what is usually considered normal and HTN, using a newly designed formula for assessing IR non-invasively. The METS-IR score was higher in those with preHTN, and there was a significant difference in BMI, TG, and HDL-c according to METS-IR quartiles. Metabolic score for insulin resistance was shown to be a single independent predictor of preHTN.

Statistically, the preHTN group had significantly higher mean values for traditional indicators of metabolic syndrome, a finding also documented in patients with established HTN (19). Both preHTN and metabolic syndrome are lifestyle conditions closely related to body weight, and they are exacerbated by excess adiposity. In preHTN, the increase in body weight affects the renin-angiotensin-aldosterone system (RAAS) regulation mechanism, while in metabolic syndrome, excess weight increases glucose levels and IR, leading to the activation of the RAAS system. This activation initiates the inflammatory process that triggers atherosclerosis and lipid dysregulation, resulting in CV diseases, especially coronary artery disease (CAD) and heart failure (HF) (20). Furthermore, most of these indicators (BMI, TG, and HDL-c) were significantly higher when compared across the quartiles of METS-IR in the preHTN group. A study conducted in Saudi Arabia documented that non-diabetic individuals newly diagnosed with either preHTN or HTN had significantly elevated TG levels (21). Regarding HDL-c, Chrusciel, et al. studied the "Associations between the lipid profile and the development of hypertension in young individuals" and concluded that there was a possible positive correlation between total HDL-c concentrations and the tendency to develop HTN, contradicting the long-standing idea of HDL-c being the "good cholesterol" (22). Chrusciel, et al. measured HDL-c subfractions in young adults and suggested that other risk factors (e.g., obesity, smoking, T2DM, and HTN) might impair HDL-c function, and high levels of large (HDL-c 3) or intermediate HDL-c may have a pro-atherogenic effect (22). Therefore, the observed high levels of HDL-c should not be considered protective in these patients.

No significant association was found between sex and quartiles of METS-IR in the preHTN group. Despite the fact that IR is generally more prevalent in men (partly due to differences in body fat distribution, with men having more visceral fat and women more subcutaneous fat), the incidence of IR in women rises and becomes more comparable to that of men after menopause. This change is partly due to estrogen, the primary female sex hormone, which appears to have protective effects on insulin sensitivity. Estrogen is partially responsible for women having better insulin sensitivity during their reproductive years (23).

In the present study, the positive correlation between METS-IR and the average BP of the two groups (r = 0.54, P < 0.001) supports the association that as the IR score increases, BP also rises from suboptimal levels, reaching the highest point in high normal BP or preHTN. This finding underscores the importance of IR as a primary factor in the development of preHTN and HTN, consistent with other studies (24, 25). It is thought that there are multiple pathways through which insulin resistance (and subsequent hyperinsulinemia) increases blood pressure beyond just activating the RAAS. Compensatory hyperinsulinemia, which results from decreased insulin sensitivity, works to maintain normal blood glucose levels by enhancing insulin-mediated glucose uptake in adipocytes and rhabdomyocytes. Additionally, hyperinsulinemia triggers increased circulating levels of norepinephrine, leading to elevated blood pressure (26). Insulin also enhances the secretion of endothelin-1 (a potent vascular constrictor), raises vascular smooth muscle calcium levels, and stimulates the RAAS, resulting in increased peripheral vascular resistance. Insulin may also raise cardiac output through its positive inotropic effect and can increase blood volume through vasopressin production (26).

Cox regression analysis showed that METS-IR is an independent predictor of preHTN. Recent studies have concluded that METS-IR is significantly associated with the risk of preHTN in euglycemic people (27, 28). This finding is also consistent with a cohort study conducted in China (29). In this cohort, Xu et al. (29) concluded that the risk of incident HTN was significantly associated with elevated IR levels (using the METS-IR score to assess insulin resistance) in individuals who are not overweight and have no abnormalities in HDL-c, TG, or FBG levels at baseline. In another cohort, Castro et al. studied the "Association of Hypertension and Insulin Resistance in Individuals Free of Diabetes in the ELSA-Brasil Cohort" and concluded that IR was a risk factor for the development of preHTN and HTN in adults who were free of diabetes and independent of weight (25).

Thus, these components of metabolic changes that may play a role in the pathophysiology of essential HTN can be regulated back to their normal values if detected early, before unrestrainable vascular stiffness occurs and chronic antihypertensive drug treatment becomes the only option (26). It is now clear that people with preHTN are not a single homogeneous group but consist of smaller subgroups. Some of these subgroups may benefit from clinical follow-up, lifestyle modification, intensified behavior control, and potentially drug treatment.

To achieve this, especially in low and middle-income countries, it is vital to adopt new tools that aid in expanding screening efforts. This is a cornerstone in strengthening PHC and helps relieve pressure on under-resourced health facilities. It is important to consider the financial value of making this tool available to developing countries, which suffer greatly from the burden of NCDs and struggling economies.

Several methods are used to assess insulin sensitivity, ranging from complex, time-consuming, and costly invasive procedures to relatively simple tests involving a single fasting blood sample. Among these methods, METS-IR fulfills the most important features of any screening test, clinical importance and suitable characteristics compared to other methods (30). According to the results of this study and those obtained from other studies, METS-IR, a novel, noninvasive, and fast method that can be easily calculated in the PHC setting—even in underdeveloped countries—can be used for screening or risk stratification and, hence, the prediction of future CV events. This makes the 10-year CVD risk effectively assessable in people with preHTN (31, 32). The increasing interest in a simple indicator for measuring IR is also highlighted in other studies (33).

This study sheds light on two important health issues: Insulin resistance and preHTN. Insulin resistance plays a crucial role in the pathophysiology of many chronic diseases and should receive significant attention in research and clinical practice, including screening. Furthermore, the diagnosis, management, and follow-up of preHTN require further attention and possibly new recommendations. To date, few studies have addressed the role of METS-IR in preHTN.

The methodology of this study, which included a matched comparison group, enabled better control for the two main confounders in the causal pathway of preHTN, namely age and sex, and in IR sexual dimorphism (34). The exclusion of individuals with a history of T2DM or abnormal glucose levels made the association between METS-IR and preHTN more independent. However, the cross-sectional design of this study was unable to determine causation, and we used the METS-IR score solely to assess insulin resistance without comparing it to the HEC (the gold standard method).

Additionally, some important confounding factors associated with elevated blood pressure were not evaluated in this study. For example, with the epidemiological transition in Iraq, sedentary lifestyles, and increasing smoking and alcohol prevalence have become essential risk factors for increased blood pressure (35).

### 5.1. Conclusions

Metabolic score for insulin resistance is significantly associated with and an independent predictor of preHTN in euglycemic people. Due to its simplicity and reliability, it can be used as an indicator for preHTN screening and risk stratification, especially in PHC settings.

## Data Availability

The authors confirm that the data supporting the findings of this study are available from the corresponding author on request.
